# Array-Based Approaches for the Identification of Epigenetic Silenced Tumor Suppressor Genes

**DOI:** 10.2174/138920208783884892

**Published:** 2008-03

**Authors:** Noriyuki Takai, Hisashi Narahara

**Affiliations:** Department of Obstetrics and Gynecology, Oita University Faculty of Medicine, Oita, Japan

**Keywords:** Tumor suppressor gene, epigenetics, DNA methylation, histone modification.

## Abstract

Carcinogenesis involves the inactivation or inhibition of genes that function as tumor suppressors. Deletions, mutations, or epigenetic silencing of tumor suppressor genes can lead to altered growth, differentiation, and apoptosis. DNA methylation and histone modifications are important epigenetic mechanisms of gene regulation and play essential roles both independently and cooperatively in tumor initiation and progression. Realization that many tumor suppressor genes are silenced by epigenetic mechanisms has stimulated discovery of novel tumor suppressor genes. One of the most useful of these approaches is an epigenetic reactivation screening strategy that combines treatment of cancer cells *in vitro* with DNA methyltransferase and/or histone deacetylase (HDAC) inhibitors, followed by global gene expression analysis using microarrays, to identify upregulated genes. This approach is most effective when complemented by microarray analyses to identify genes repressed in primary tumors. Recently, using cancer cell lines treated with a DNA methylation inhibitor and/or a HDAC inhibitor in conjunction with cDNA microarray analysis, candidate tumor suppressor genes, which are subject to epigenetic silencing, have been identified in endometrial, colorectal, esophageal, and pancreatic cancers. An increasing number of studies have utilized epigenetic reactivation screening to discover novel tumor suppressor genes in cancer. The results of some of the most recent studies are highlighted in this review.

## INTRODUCTION

Many cancers have been characterized by multiple genetic and epigenetic alterations [1, 2]. In cancer cells, aberrant methylation of CpG islands has been found in the 5’ end of the regulatory regions and/or the first exons of tumor suppressor genes and genes responsible for genomic stability [3, 4]. Epigenetic changes can silence the transcription of these important genes, leading to clonal proliferation of tumor cells [3, 4]. Epigenetic modifications of cytosine residues in DNA and the NH2 termini of histone proteins have emerged as key mechanisms in chromatin remodeling, affecting transcriptional regulation. Growing evidence exists that interplay occurs between cytosine methylation and histone modification. The methyl-CpG binding protein, MeCP2, has been found to be associated with histone deacetylase (HDAC) activity, providing a pathway by which histone modification can be induced by DNA methylation changes [4-6]. The identification and characterization of genes whose CpG islands in the promoter are selectively hypermethylated in cancers not only improves our understanding of the role of epigenetic alterations in tumorigenesis but may also lead to the discovery of novel tumor suppressor genes. Recently, using cancer cell lines treated with a DNA methylation inhibitor and/or a HDAC inhibitor in conjunction with cDNA microarray analysis, candidate tumor suppressor genes, which are subject to epigenetic silencing, have been identified in endometrial [2], colorectal [7], esophageal [8], and pancreatic [9] cancers.

## ENDOMETRIAL CANCERS

**Fig. (1):**
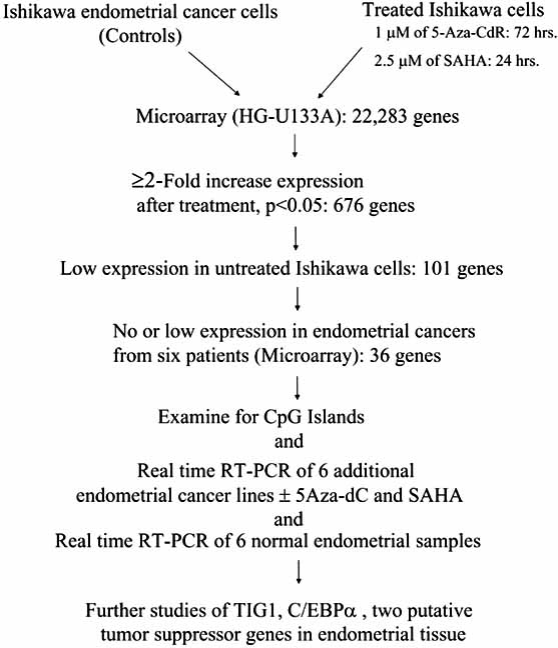
Outline of selection of tumor suppressor genes in endometrial tissue.

Takai *et al*. [2] used the cDNA microarray technique to identify genes significantly (*P* < 0.05) up-regulated in an endometrial cancer cell line, Ishikawa, after treatment with 5-aza-2’-deoxycytidine (5-Aza-CdR; 3 days), which blocks DNA methylation, and suberoylanilide bishydroxamide (SAHA; 1 day) to inhibit HDAC. The gene expression profile was compared before and after treatment using Affymetrix (Santa Clara, CA) human genome U133A microarray chips containing 22,283 transcripts (Fig. **1**). Treatment with these agents resulted in up-regulation (defined as a ≥2.0-fold increase) of 676 genes. They focused on 101 genes that were either not expressed or expressed only at low levels (raw values <500) before treatment of the cell line. To narrow further the list of candidate genes, they queried their expression status in six endometrial cancer samples as determined by microarray analysis. The clinical samples were individually analyzed by microarray. Thirty-six of the 101 genes were expressed at very low levels in the clinical cancer samples. The 5’ regulatory regions of these genes were analyzed in the BLAST database and the CpG Island Searcher [10] to determine whether they contained CpG islands. Thirty-two of the 36 (89%) genes harbored CpG sites in the promoter region.

Expression of these 36 genes was examined in six endometrial cancer cell lines, including Ishikawa, with or without 5-Aza-CdR and SAHA using real-time quantitative reverse transcription-PCR (RT-PCR). The *Tig1* and *C/ebpa* genes were completely silenced in all six endometrial cancer cell lines, and their expressions were induced in each cell line after exposure to 5-Aza-CdR and SAHA.

**Fig. (2):**
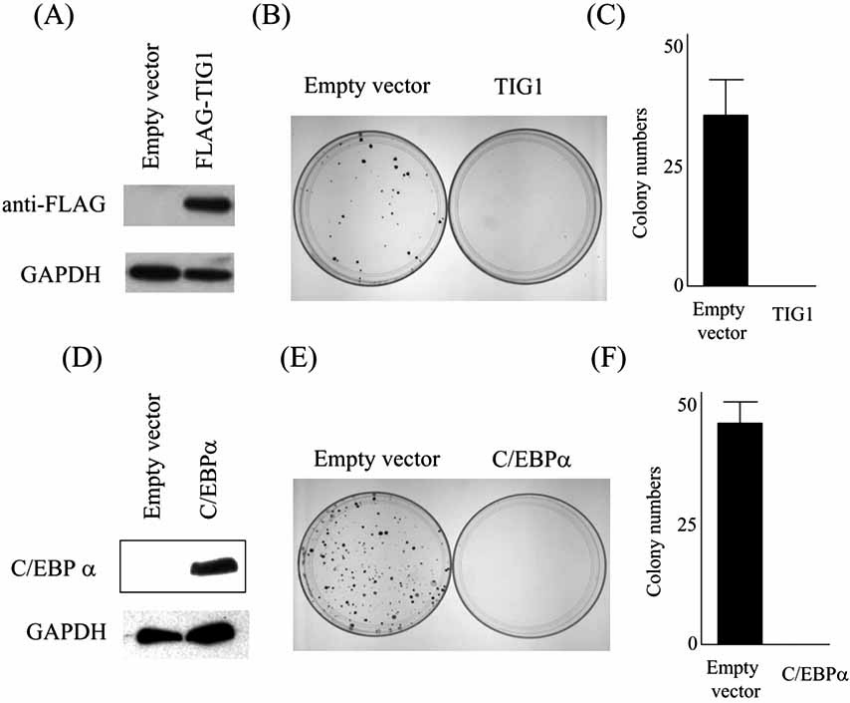
Enforced expression of either *Tig1* or *C/ebpa* suppressed clonogenic growth of Ishikawa cells. Ishikawa endometrial cancer cells were transfected with either pcDNA3.1 (Empty vector), pcDNA3-FLAG-*Tig1* (TIG), or pcDNA3-*C/ebpa* (C/EBPα). Protein expression levels at 24 hours after transfection were confirmed by Western blot using either anti-FLAG or anti-C/EBPα antibody (A and D). Transfected cells were selected with G418, and resistant colonies (after 14 days of culture in G418) were stained with crystal violet (B and E). The numbers of the colonies were counted (C and F). Columns, mean of experiments repeated thrice with triplicate dishes per experimental point; bars, SD.

Expression of both genes was low in endometrial cancer cell lines and clinical samples but high in normal endometrial tissues. Bisulfite sequencing, restriction analysis, and/or methylationspecific PCR revealed aberrant methylation of the CpG island in the *Tig1* gene of all 6 endometrial cancer cell lines examined and 4 of 18 clinical endometrial cancers, whereas the *C/ebpa* promoter remained unmethylated in endometrial cancers. Chromatin immunoprecipitation showed increased acetylated histone H3 bound to both *Tig1* and *C/ebpa* genes after treatment with 5-Aza-CdR and/or SAHA. Forced expression of either TIG1 or C/EBPα led to significant growth reduction of Ishikawa cells (Fig. **2**).

Their data suggest that TIG1 or C/EBPα function as tumor suppressor proteins in endometrial cancers and that their reexpression may be a therapeutic target.

## COLORECTAL CANCERS

Suzuki *et al*. [7] used cDNA microarray technology to identify genes upregulated in the colorectal cancer (CRC) cell line RKO, after cells were treated with low-dose 5-Aza-CdR, which minimally blocks DNA methylation, and trichostatin A (TSA) to inhibit HDAC. Of a total of 10,814 genes examined by subtraction microarray, 74 were upregulated by treatment with 5-Aza-CdR and/or TSA. Of all of the non-EST (expressed sequence tags) genes, 56 had known chromosomal positions. They were also able to identify 5' CpG islands (GC content <60%, ratio of CpG to GpC <0.6 and minimum length 200 bp) for 27 of the 56 genes.

Their findings suggested that *SFRP1*, *SEZ6L*, *CXX1*, *KIAA0786*, *S100A10* and *TIMP2* might influence tumor development and progression. They therefore studied these genes in tumor cell lines of other types of cancer. A pattern of tumor profiling emerged: complete hypermethylation of *SFRP1*, *SEZ6L*, *LPPH1* and *CXX1* was common in CRC and gastric cancers, but only partial or no methylation was seen in all other types of cancer studied. They found exceptions to this pattern for *SFRP1*. This gene has been shown to induce apoptosis in a breast cancer cell line, MCF7, which did not express the gene in the basal state [11]. They found complete methylation of the CpG island region in this cell line, as well as in MDA MB231 breast cancer cells and two of four prostate cancer cell lines studied.

## ESOPHAGEAL CANCERS

Yamashita *et al*. [8] performed a comprehensive survey of commonly inactivated tumor suppressor genes in esophageal squamous cell carcinoma (ESCC) based on functional reactivation of epigenetically silenced tumor suppressor genes by 5-Aza-CdR and TSA using microarrays containing 12,599 genes. Among 58 genes identified by this approach, 44 (76%) harbored dense CpG islands in the promoter regions. Thirteen of twenty-two tested gene promoters were methylated in cell lines, and ten in primary ESCC accompanied by silencing at the mRNA level. Potent growth suppressive activity of three genes including *CRIP-1*, *Apolipoprotein D*, and *Neuromedin U* in ESCC cells was demonstrated by colony focus assays.

## PANCREATIC CANCERS

To identify potential targets for aberrant methylation in pancreatic cancer, Sato *et al*. [9] analyzed global changes in gene expression profiles of four pancreatic cancer cell lines after treatment with the demethylating agent 5-Aza-CdR and/or the histone deacetylase inhibitor TSA. A substantial number of genes were induced 5-fold or greater by 5-Aza-CdR alone (631 transcripts), TSA alone (1196 transcripts), and by treatment with both agents (857 transcripts). Four hundred and seventy-five genes were markedly (>5-fold) induced after 5-Aza-CdR treatment in pancreatic cancer cell lines but not in a nonneoplastic pancreatic epithelial cell line. The methylation status of 11 of these 475 genes was examined in a panel of 42 pancreatic cancers, and all 11 of these genes were aberrantly methylated in pancreatic cancer but rarely, if any, methylated in 10 normal pancreatic ductal epithelia. These genes include *UCHL1* (methylated in 100% of 42 pancreatic cancers), *NPTX2* (98%), *SARP2* (95%), *CLDN5* (93%), *reprimo* (86%), *LHX1* (76%), *WNT7A* (71%), *FOXE1* (69%), *TJP2* (64%), *CDH3* (19%), and *ST14* (10%). Three of these 11 genes (*NPTX2*, *SARP2*, and *CLDN5*) were selected for further analysis in a larger panel of specimens, and aberrant methylation of at least one of these three genes was detectable in 100% of 43 primary pancreatic cancers and in 18 of 24 (75%) pancreatic juice samples obtained from patients with pancreatic cancer. Thus, a substantial number of genes are induced by 5-Aza-CdR treatment of pancreatic cancer cells, and many of them may represent novel targets for aberrant methylation in pancreatic carcinoma.
